# Preoperative Factors Associated with Infiltrative Histologic Growth Patterns in Extremity Soft Tissue Sarcoma

**DOI:** 10.1155/2017/5419394

**Published:** 2017-07-20

**Authors:** Jong Woong Park, Han-Soo Kim, Cheol Lee, Hye Jin Yoo, Ji Yeon Yun, Ilkyu Han

**Affiliations:** ^1^Department of Orthopaedic Surgery, Seoul National University Hospital, Seoul, Republic of Korea; ^2^Department of Pathology, Seoul National University College of Medicine, Seoul, Republic of Korea; ^3^Department of Radiology, Seoul National University Hospital, Seoul, Republic of Korea

## Abstract

Soft tissue sarcoma (STS) with an infiltrative histologic growth pattern, when compared to STS with an expansile pattern, may pose difficulties in local control. Preoperative assessment of the presence of infiltrative histologic growth pattern would be helpful in deciding treatment strategies. A review of 144 patients who underwent surgery for extremity STS was performed. Microscopically, the histologic growth pattern was defined as infiltrative if the penetration of the tumor cells into the surrounding tissue was observed. Possible clinicopathologic factors that might be associated with infiltrative histologic growth pattern were investigated with regard to patient demographics, tumor characteristics, and MRI findings. Of the 144 tumors, 71 (49%) showed infiltrative histologic growth pattern. On multivariate analysis, histological subtypes other than liposarcoma (OR = 4.57, *p* = 0.02) and infiltrative border on MRI (OR = 2.48, *p* = 0.01) were independent factors associated with infiltrative histologic growth pattern. Predictive index based on these two factors showed a significant improved accuracy (ROC-AUC = 0.647) for predicting infiltrative histologic growth pattern compared to either factor alone. Our data suggests that liposarcoma histology and tumor border on MRI can predict histologic growth pattern in extremity STS.

## 1. Introduction

*Local recurrence in extremity soft tissue sarcomas (STS)* not only is associated with poor oncologic outcome but also has deleterious effects on limb function [1–4]. Local recurrence is largely dictated by the ability to achieve histologically negative resection margins, and failure to obtain histologically negative margins represents microscopic residual disease and translates into high rates of local recurrence [5, 6]. To obtain histologically negative resection margin, accurate determination of histologic tumor extent is necessary.

Histologic growth pattern has been associated with local recurrence in extremity STS [7–9]. STS with an infiltrative histologic growth pattern, when compared to STS with an expansile growth pattern, may pose difficulties in local control and subsequent risk for local recurrence [10, 11]. Thus, preoperative assessment of the infiltrative histologic growth pattern, using preoperatively available clinicopathologic parameters, would be helpful in guiding surgery and adjuvant treatment. The purpose of this study was to identify clinicopathologic characteristics that are predictive of infiltrative histologic growth pattern in extremity STS.

## 2. Material and Methods

### 2.1. Patient Selection

From our institutional database, 289 patients who underwent curative surgery for extremity STS between 2009 and 2014 were retrospectively reviewed. For the purpose of analysis, 109 patients in whom histologic growth pattern of the entire tumor periphery could not be evaluated were excluded. Of the remaining 180 patients, patients with well-differentiated liposarcoma (*n* = 24), patients for whom histological grade could not be evaluated (*n* = 10), and patients without MRI (*n* = 2) were also excluded, which left 144 patients for analysis. The institutional review board of our institute approved this study.

### 2.2. Assessment of Histologic Growth Pattern

**Figure 1:**
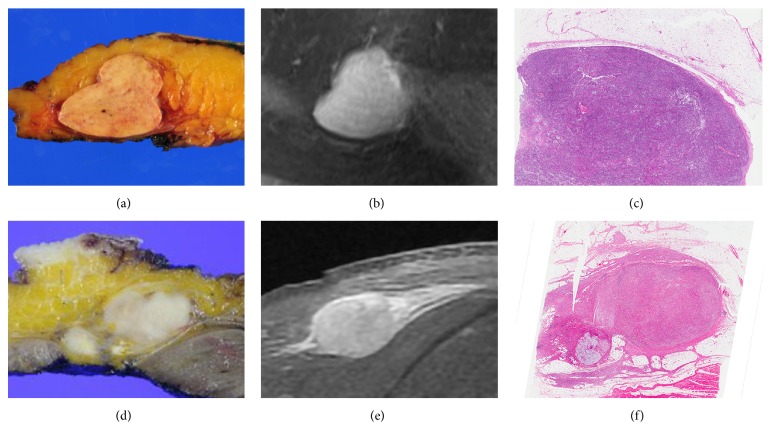
Representative sections of histologic growth pattern. Photomicrograph and MRI ((a) gross, (b) gadolinium-enhanced T1-weighted sequences with fat suppression, and (c) H&E staining, magnification ×1) of the histological specimen of an subcutaneous synovial sarcoma with expansile growth pattern. Photomicrograph ((d) gross, (e) gadolinium-enhanced T1-weighted sequences with fat suppression, and (f) H&E staining, magnification ×1) of the histological specimen of a subcutaneous undifferentiated pleomorphic sarcoma with an infiltrative growth pattern.

The cut surface of the whole tumor was obtained from the maximum diameter and was embedded into paraffin after which 4-*μ*m sections were made. The microscopic assessment was performed on 4-*μ*m sections stained with hematoxylin-eosin. The cut surface was examined, and several representative sections were made encompassing the tumor and surrounding tissue. Microscopically the histologic growth pattern was defined as infiltrative if the penetration of the tumor cells into the surrounding tissue was observed (Figure 1(d)). Focal penetration of the tumor cells was regarded as infiltrative growth pattern. Histologic growth pattern was defined as expansile if the penetration of the tumor cells was not observed regardless of the presence of the pseudocapsule (Figure 1(a)) [10, 12].

### 2.3. Analyses of Factors Predictive of Infiltrative Histologic Growth Pattern

Medical records were reviewed for the potential radiological and clinical factors that might be predictive of infiltrative histologic growth pattern in STS: (1) patient demographics, (2) tumor characteristics, and (3) radiological characteristics. For patient demographics, gender, age, presentation status, and history of adjuvant therapy were investigated. There were 63 women (44%) and 81 men (56%). The mean age at the time of STS diagnosis was 50 years (range, 5–86 years). Twenty-two patients (15%) presented with locally recurrent tumors and 21 patients (15%) presented after an unplanned removal of a STS. Twenty patients (14%) had metastatic disease at the time of diagnosis of STS. STS were resected with wide margin in 125 cases (87%), marginal margin in 18 cases (12%), and intralesional margin in 1 case (1%). Pathologically negative margins were achieved in 131 patients (90%). Radiation therapy was administered in 83 patients (57%). All patients received external beam radiation and the median dose was 60 Gy (range, 50–65 Gy). Chemotherapy was administered in 28 patients (20%) with 10 patients (7%) receiving preoperative chemotherapy (Table 1).

For tumor characteristics, tumor location, histologic diagnosis, histologic grade, tumor size, and tumor depth were investigated. STS were commonly located at thigh (*n* = 61, 42%), lower leg (*n* = 19, 13%), knee (*n* = 13, 9%), buttock (*n* = 20, 14%), shoulder (*n* = 11, 8%), upper arm (*n* = 9, 6%), forearm (*n* = 5, 4%), and trunk wall (*n* = 6, 4%). Most common histological diagnoses were undifferentiated pleomorphic sarcoma (UPS, *n* = 26, 18%), synovial sarcoma (*n* = 23, 16%), myxofibrosarcoma (*n* = 22, 15%), liposarcoma (*n* = 17, 12%), and leiomyosarcoma (*n* = 14, 10%) (Supplementary Table  1 in Supplementary Material available online at https://doi.org/10.1155/2017/5419394). Patients with well-differentiated liposarcoma were excluded. As for histologic grading, there were 23 grade 1 (16%), 54 grade 2 (38%), and 67 grade 3 (46%) tumors according to the Federation Nationale des Centres de Lutte Contre le Cancer (FNCLCC) classification system [13]. Mean size of the primary tumor, measured by the largest diameter on preoperative MRI, was 7.7 cm (range, 2.0–42.5 cm). For tumor depth, tumors located above the superficial fascia were defined as superficial. Twenty-one tumors (15%) were superficial and 123 tumors were deep seated (85%) (Table 1).

**Figure 2:**
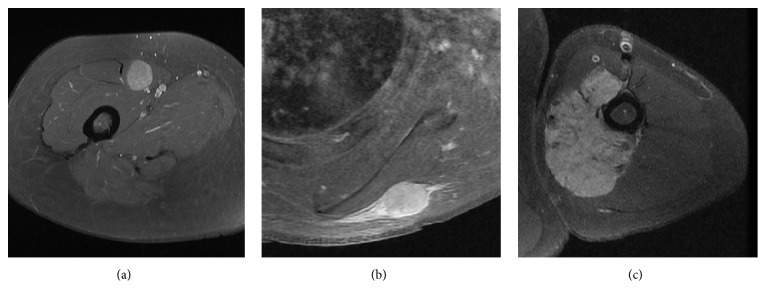
Representative images of different tumor borders on MRI. (a) T1 enhanced MRI of an intramuscular synovial sarcoma with pushing border on MRI. (b) T1 enhanced MRI of an intramuscular undifferentiated pleomorphic sarcoma with infiltrative border on MRI with the tail sign. (c) T1 enhanced MRI of a leiomyosarcoma with infiltrative border on MRI without the tail sign.

For radiological characteristics, compartmental status [14], presence of peritumoral edema [15, 16], and the tumor border [7] were investigated using preoperative MRI scans. Spin-echo T1-weighted sequences, fast spine-echo T2-weighted sequences with fat suppression, and gadolinium-enhanced T1-weighted sequences with fat suppression were available in all MRIs. Gadolinium-enhanced T1-weighted sequences were obtained in at least two orthogonal planes. MRI scans were performed with a 1.5- or 3-T system. MRI images were reviewed by two orthopaedic oncologists (J. W. P and I. H) and a radiologist (H. J. Y) with expertise in musculoskeletal imaging. Compartmental status was defined as intracompartmental or extracompartmental as described by Enneking et al. [14]. There were 91 intracompartmental (63%) and 53 extracompartmental tumors (37%). Peritumoral edema was defined as diffuse regions of increased T2-weighted signal intensity surrounding the tumor [15, 16]. Of the 144 tumors, 39 tumors (27%) showed peritumoral edema. Tumor border on MRI was assessed on T2-weighted and enhanced T1-weighted images. Pushing border was defined as a well-defined border without peripheral extension to the surrounding tissue (Figure 2(a)), while infiltrative border was defined as an irregular border with spicula-like extensions into the surrounding tissue (Figures 2(b) and 2(c)) [7, 17–21]. Infiltrative border on MRI was identified as two different patterns: the first pattern with the tail sign, defined as a curvilinear shaped tapered thick fascial enhancement extending from the primary mass, with or without irregularity of tumor border (Figure 2(b)) [8, 17–20]; the second pattern with irregular or spiculated borders extending to surrounding tissue without the tail sign (Figure 2(c)). Of the 144 tumors, 87 tumors (60%) had infiltrative border and 57 (40%) had pushing border. Of the 87 tumors with infiltrative border, 68 tumors showed the tail sign while 19 tumors showed infiltrative border without the tail sign (Table 1).

### 2.4. Statistical Analyses

Continuous measures were compared using the independent-samples *t*-test and categorical variables were compared using Pearson's chi-squared test. Clinical or radiological factors which were found to have a statistically significant association with the histologic growth pattern (*p* < 0.05) were included in a multivariate logistic regression analysis, with backward selection using the likelihood ratio test, to evaluate associations linking the histologic growth pattern. Statistical analysis was performed using SPSS v.21.0 software (IBM Inc., Armonk, New York). To test the predictive accuracy of identified variables, receiver-operating characteristic (ROC) curves were constructed, and the area under the curve (AUC) with a 95% confidence interval was calculated. Pairwise AUC comparisons were also performed between the two variables at using the nonparametric approach developed by DeLong et al. [22]. For all statistical comparisons, a *p* value of 0.05 was considered statistically significant.

## 3. Results

### 3.1. Comparison of Characteristics between Patients with Infiltrative Histologic Growth Pattern and Those with Expansile Histologic Growth Pattern

Of the 144 tumors, 71 (49%) showed histologic infiltrative growth pattern. STS with infiltrative histologic growth pattern were more likely to be superficial (*p* = 0.03) and to show infiltrative tumor border on MRI (*p* = 0.01) (Table 2). Among the preoperative factors analyzed, there were no significant difference found with respect to age, gender, administration of preoperative radiation therapy or chemotherapy, tumor size, compartmental status, presentation status, or presence of peritumoral edema on MRI. STS with infiltrative histologic growth pattern showed trend towards having higher chance of pathologically positive margins and local recurrence (Table 2).

### 3.2. Factors Associated with Infiltrative Histologic Growth Pattern

On univariate logistic regression analysis of associated factors of infiltrative histologic growth pattern in STS, superficial location (*p* = 0.03), infiltrative tumor border on MRI (*p* = 0.01), and histologic subtype other than liposarcoma (*p* = 0.01) were significant (Table 3). On multivariate analysis, infiltrative tumor border on MRI (OR = 2.48, *p* = 0.01) and histological subtype other than liposarcoma (OR = 4.57, *p* = 0.02) remained as independent factors associated with infiltrative histologic growth pattern (Table 3).

### 3.3. Predictive Accuracy of Identified Factors

To test the accuracy of predicting the presence of infiltrative histologic growth pattern, we generated a predictive index based on the presence or absence of two significant factors found in the multivariate analysis. Cases with both histological subtype other than liposarcoma and infiltrative tumor border on MRI were considered to have positive predictive index. All other cases were considered to have negative predictive index.

**Figure 3:**
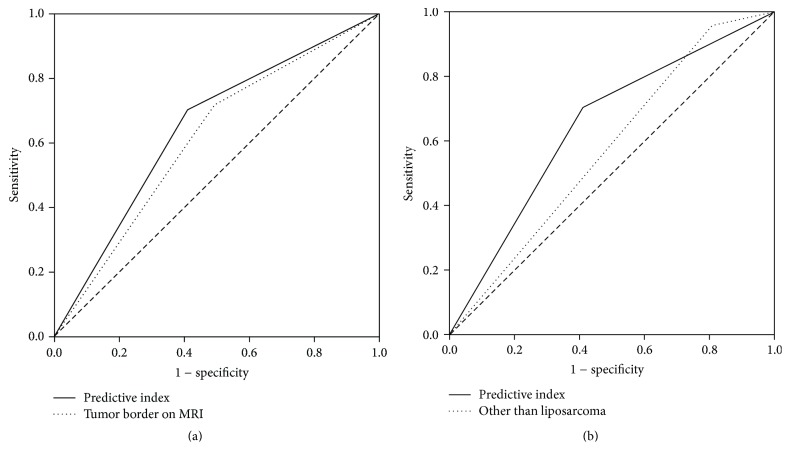
Pairwise AUC comparison of the predictive index and the tumor border on MRI alone or histological subtype other than liposarcoma alone in predicting infiltrative histologic growth pattern. (a) Pairwise area under the receiver-operating curve (AUC) comparison of the predictive index and the tumor border on MRI alone in predicting infiltrative histological growth pattern. (b) Pairwise area under the receiver-operating curve (AUC) comparison of the predictive index and histological subtype other than liposarcoma alone in predicting infiltrative histological growth pattern. AUC, area under the curve. The dashed line in both graphs represented random guess to predict (AUC = 0.500).

ROC curves yielded an AUC of 0.613 (95% CI = 0.520–0.705) for the tumor border on MRI, 0.575 (95% CI = 0.481–0.668) for the histological subtype other than liposarcoma, and 0.647 (95% CI = 0.556–0.737) for the predictive index. The improvement of AUC by the predictive index was significant in comparison with infiltrative tumor border on MRI alone (*p* = 0.010) (Figure 3(a)) or histological subtype other than liposarcoma alone (*p* = 0.028) (Figure 3(b)).

## 4. Discussion

Infiltrative histologic growth pattern has been associated with poor oncologic outcome, not only for local control but also for survival in extremity STS [7, 8]. Preoperative assessment of infiltrative histologic growth pattern would be helpful in planning treatment of extremity STS. This study examined various preoperative clinicopathologic factors and identified nonliposarcoma histology and infiltrative tumor border on MRI as independent predictors of infiltrative histologic growth pattern. To our knowledge, this is the first study to examine various preoperative factors that can predict histologic growth pattern in extremity STS.

A few things should be considered while interpreting the results of this study. First, this study was based on retrospective review of a patient cohort from a single tertiary referral hospital with relatively small number of patients. The results of this study need to be validated in external databases in a prospective setting. Second, as for the evaluation of MRI characteristics, the MRI protocols were not uniform because of the retrospective nature of the study. Third, in our routine practice, the pathologic findings according to the specific radiological abnormalities were not evaluated, and such radiological–pathological correlation could not be performed due to the retrospective nature of the study. Fourth, some of the factors might not be accurately assessed preoperatively, such as the histological subtype or histological grade, especially in cases where core needle biopsy is used. Fifth, the limited use of recently developed biomarkers, such as MDM2, may hamper the accurate diagnosis of histological subtype.

In this study, larger tumor size was not associated with infiltrative histological growth pattern. The authors excluded well-differentiated liposarcomas, which often present with large tumor size, despite having a less aggressive biology. However, the association between tumor size and histological growth pattern remained insignificant even after the exclusion of well-differentiated liposarcomas.

Histological subtypes of STS seems to be associated with histologic growth pattern. Majority of STS subtypes showed predominantly infiltrative histologic growth pattern; UPS (62%, 16/26), myxofibrosarcoma (59%, 13/22), and synovial sarcoma (57%, 13/23). However, predominantly expansile histologic growth pattern was observed in MPNST (87%, 7/8) and liposarcoma (82%, 14/17). As current treatment strategies for STS are increasingly adapted to a specific histological subtype, predictive factors for specific subtypes of STS will be needed.

The value of tumor border on MRI in predicting histologic growth pattern seem to differ among histological subtypes of STS. The association of infiltrative tumor border on MRI, most notably the tail sign, with infiltrative histologic growth pattern has been well-documented in UPS and myxofibrosarcoma [7, 17–20, 23]. Indeed, tumor border on MRI predicted infiltrative histologic growth pattern in UPS (AUC = 0.619, 95% CI = 0.385–0.852) and myxofibrosarcoma (AUC = 0.649, 95% CI = 0.391–0.907) with moderate accuracy in this study. However, for synovial sarcoma, ROC curve yielded an AUC of 0.519 (95% CI = 0.276–0.762). These data suggest that the predictive value of tumor border on MRI in assessing histologic growth pattern differs among histologic subtypes of STS.

Histologic growth patterns may be affected by the tumor environment. Tumors located in the superficial location, where no fascial boundaries exist, had more tumors with infiltrative histologic growth pattern than tumors located in the deep location in the univariate analysis. Moreover, tumors presenting with previous unplanned surgery or local recurrence, in which the normal anatomical boundaries are violated, had more tumors with infiltrative histologic growth pattern than tumors presenting without any previous surgeries. Taken together, histologic growth pattern seems to be affected not only by the tumor biology but also by the tumor environment.

The predictive accuracy of the predictive index generated in this study was at best moderate with an AUC value of 0.647. Better predictive factors, such as genomic markers, are needed to improve predicting the histologic growth pattern in extremity STS. However, the results of this study can be implemented easily in the clinical practice and may provide a backbone for newly identified predictive factors of histologic growth pattern in extremity STS.

## 5. Conclusions

In conclusion, our data suggests that liposarcoma histology and tumor border on MRI can predict histologic growth pattern in extremity STS. If an extremity STS of nonliposarcoma histology shows infiltrative tumor border on MRI, infiltrative histologic growth pattern can be expected.

## Supplementary Material

Soft tissue sarcoma histologic types.

## Figures and Tables

**Table 1:** Preoperative characteristics.

Patient characteristics	
Mean age (yrs, SD^a^)	50.0 (19.7)
Gender (*n*, %)	
Female	63 (44)
Male	81 (56)
Presentation status (*n*, %)	
Fresh	101 (70)
Recurred	22 (15)
Unplanned excision	21 (15)
Radiation therapy (*n*, %)	
Preoperative	1 (1)
Postoperative	82 (57)
Not done	61 (42)
Dose of radiation therapy (Gy, range)	60 (50–65)
Chemotherapy (*n*, %)	
Preoperative	3 (2)
Postoperative	18 (13)
Pre- and postoperative	7 (5)
Not done	116 (80)

Tumor characteristics	

Size (cm, SD)	7.7 (6.4)
Depth (*n*, %)	
Superficial	21 (15)
Deep	123 (85)
Compartmental status (*n*, %)	
Intracompartmental	91 (63)
Extracompartmental	53 (37)
FNCLCC grade (*n*, %)	
1	23 (16)
2	54 (38)
3	67 (46)
Histologic type (*n*, %)	
UPS^b^	26 (18)
Synovial sarcoma	23 (16)
Myxofibrosarcoma	22 (15)
Liposarcoma	17 (12)
Leiomyosarcoma	14 (10)
Others	42 (29)

MRI characteristics	

Peritumoral edema (*n*, %)	
Absent	105 (73)
Present	39 (27)
Tumor border on MRI (*n*, %)	
Pushing	57 (40)
Infiltrative	87 (60)

^a^SD, standard deviation; ^b^UPS; undifferentiated pleomorphic sarcoma.

**Table 2:** Comparison of preoperative characteristics by histological growth pattern.

	Expansile	Infiltrative	*p* value
Mean age (years, SD^a^)	48 (20)	52 (20)	0.14
Gender (*n*)			0.52
Female	30	33	
Male	43	38	
Preoperative radiation therapy (*n*)			0.49
Done	0	1	
Not done	73	70	
Preoperative chemotherapy (*n*)			0.53
Done	4	6	
Not done	69	65	
Depth (*n*)			0.03
Superficial	6	15	
Deep	67	56	
Size (cm, SD)	7.7 (5.1)	7.6 (7.5)	0.91
Compartmental status (*n*)			0.52
Intracompartmental	48	43	
Extracompartmental	25	28	
Presentation status (*n*)			0.20
Fresh	56	45	
Recurred	8	14	
Unplanned excision	9	12	
Peritumoral edema (*n*)			0.65
Absent	52	53	
Present	21	18	
Tumor border on MRI (*n*)			0.01
Pushing	37	20	
Infiltrative	36	51	
FNCLCC grade (*n*)			0.83
1	13	10	
2	27	27	
3	33	34	
Surgical margin (*n*)			0.32
Wide	66	59	
Marginal	7	11	
Intralesional	0	1	
Pathologic margin (*n*)			0.35
Negative	68	63	
Positive	5	8	
Local recurrence (*n*)			0.37
No	59	53	
Yes	14	18	
Metastasis (*n*)			0.56
No	46	48	
Yes	27	23	

^a^SD, standard deviation.

**Table 3:** Preoperative factors associated with histological growth pattern.

Factors	Univariate			Multivariate		
	OR^a^	95% CI^b^	*p* value	OR	95% CI	*p* value
Age	1.01	1.00–1.03	0.14			
Gender			0.80			
Female	1.00					
Male	0.80	0.41–1.55				
Preoperative radiation therapy			1.00			
Done	1.00					
Not done	1.00					
Preoperative chemotherapy			0.49			
Done	1.59	0.43–5.90				
Not done	1.00					
Depth			0.03^c^			
Superficial	3.00	1.09–8.22				
Deep	1.00					
Size	1.00	0.95–1.05	0.91			
Compartmental status			0.51			
Intracompartmental	1.00					
Extracompartmental	1.25	0.63–2.46				
Previous treatment			0.20			
Fresh	1.00					
Recurred	2.18	0.84–5.65				
Unplanned excision	1.66	0.64–4.29				
Peritumoral edema			0.65			
Absent	1.00					
Present	0.84	0.40–1.76				
Tumor border on MRI			0.01^c^			0.01^c^
Pushing	1.00			1.00		
Infiltrative	2.62	1.31–5.23		2.48	1.21–5.08	
FNCLCC grade			0.83			
1	1.00					
2	1.30	0.49–3.47				
3	1.34	0.52–3.48				
Liposarcoma			0.01^c^			0.02^c^
Liposarcoma	1.00			1.00		
Others	5.38	1.47–19.63		4.57	1.22–17.05	

^a^OR, odds ratio; ^b^CI, confidence interval; ^c^statistically significant.

## Additional Points

Our data suggest that patients with histological diagnosis other than liposarcoma and the presence of infiltrative border on MRI can predict infiltrative histologic growth pattern in extremity soft tissue sarcoma.
