# Supplementary material for: Preoperative Factors Associated with Infiltrative Histologic Growth Patterns in Extremity Soft Tissue Sarcoma
Source: Sarcoma. 2017 Jul 20;2017:5419394. doi: 10.1155/2017/5419394 (PMC5541793; doi:10.1155/2017/5419394)
Supplement: Supplementary file 1 — Soft tissue sarcoma histologic types. [file 5419394.f1.docx]

Supplementary Table 1. List of histologic types

| **Histologic type** | **n (%)** |
| --- | --- |
| Undifferentiated pleomorphic sarcoma | 26 (18) |
| Synovial sarcoma | 23 (16) |
| Myxofibrosarcoma | 22 (15) |
| Liposarcoma | 17 (12) |
| Leiomyosarcoma | 14 (10) |
| Extraskeletal chondrosarcoma | 8 (6) |
| MPNST | 8 (6) |
| Fibrosarcoma | 6 (4) |
| Rhabdomyosarcoma | 5 (3) |
| Extraskeletal osteosarcoma | 3 (2) |
| Epithelioid sarcoma | 3 (2) |
| Clear cell sarcoma | 2 (1) |
| Low grade fibromyxoid tumor | 2 (1) |
| Malignant rhabdoid tumor | 1 (1) |
| Extraskeletal Ewing sarcoma | 1 (1) |
| Alveolar soft part sarcoma | 1 (1) |
| Angiosarcoma | 1 (1) |
| Malignant hemangiopericytoma | 1 (1) |
